# Supplementary figures and images for: Structural insights into Cullin4-RING ubiquitin ligase remodelling by Vpr from simian immunodeficiency viruses
Source: PLoS Pathog. 2021 Aug 2;17(8):e1009775. doi: 10.1371/journal.ppat.1009775 (PMC8360603; doi:10.1371/journal.ppat.1009775)

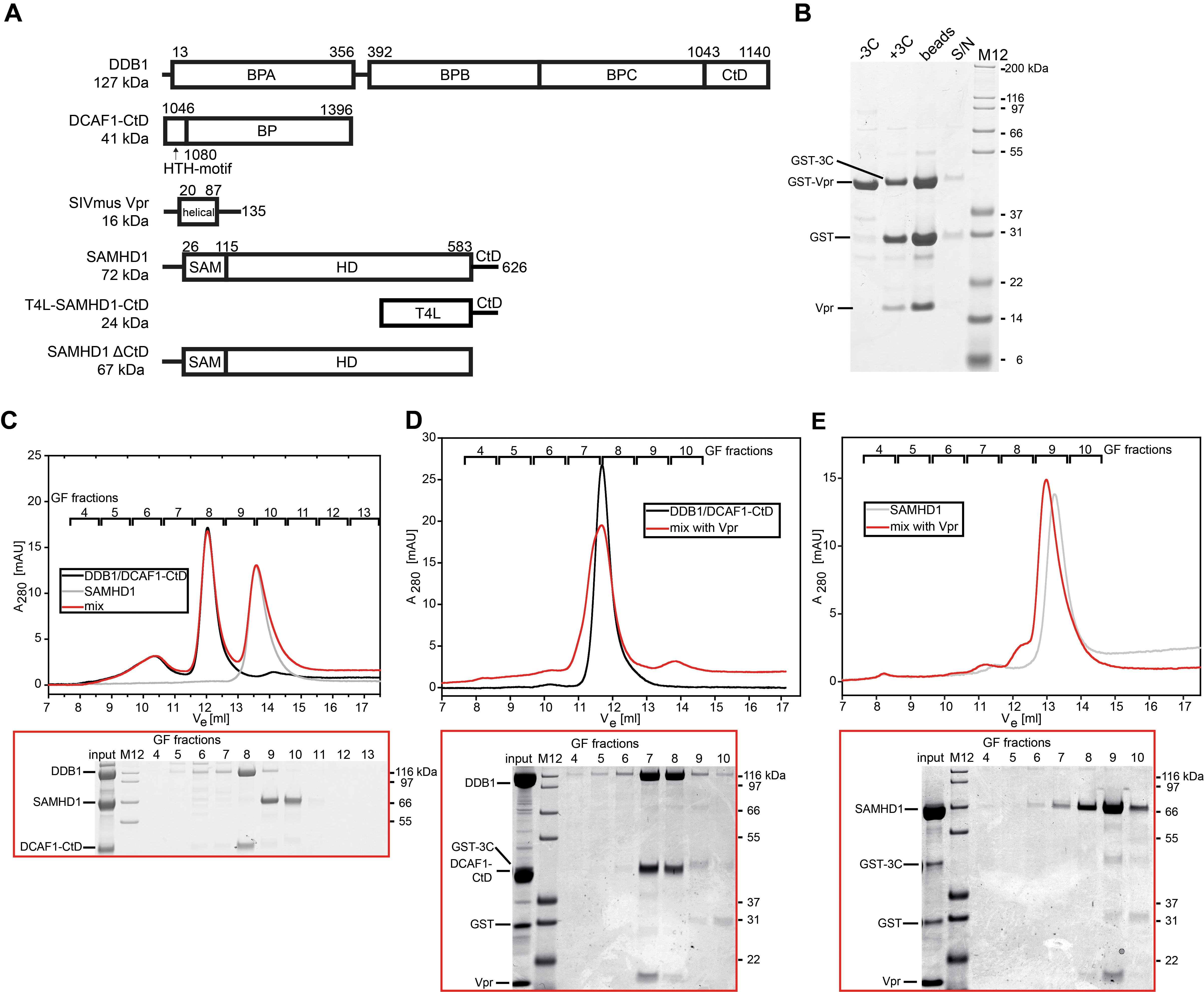

Supplement: S1 Fig — (A) Schematic view of the protein constructs used in biochemical analyses. BP–β-propeller domain, HD–histidine-aspartate domain, HTH–helix-turn-helix motif, SAM–sterile alpha motif. (B) SDS-PAGE analysis of GST-Vprmus. After treatment with 3C protease to remove the GST-tag (+3C) and GSH-Sepharose pull down to remove protease and tag, no Vprmus is present in the eluted fraction (S/N) indicating that it interacts non-specifically with the GSH-Sepharose beads and/or becomes insoluble after tag removal. (C-E) Analytical GF analysis of DDB1/DCAF1-CtD incubated with SAMHD1 (C), DDB1/DCAF1-CtD incubated with Vprmus (D) and SAMHD1 incubated with Vprmus (E). SDS-PAGE of the corresponding GF fractions is shown below each chromatogram. (TIF) [file ppat.1009775.s001.tif]

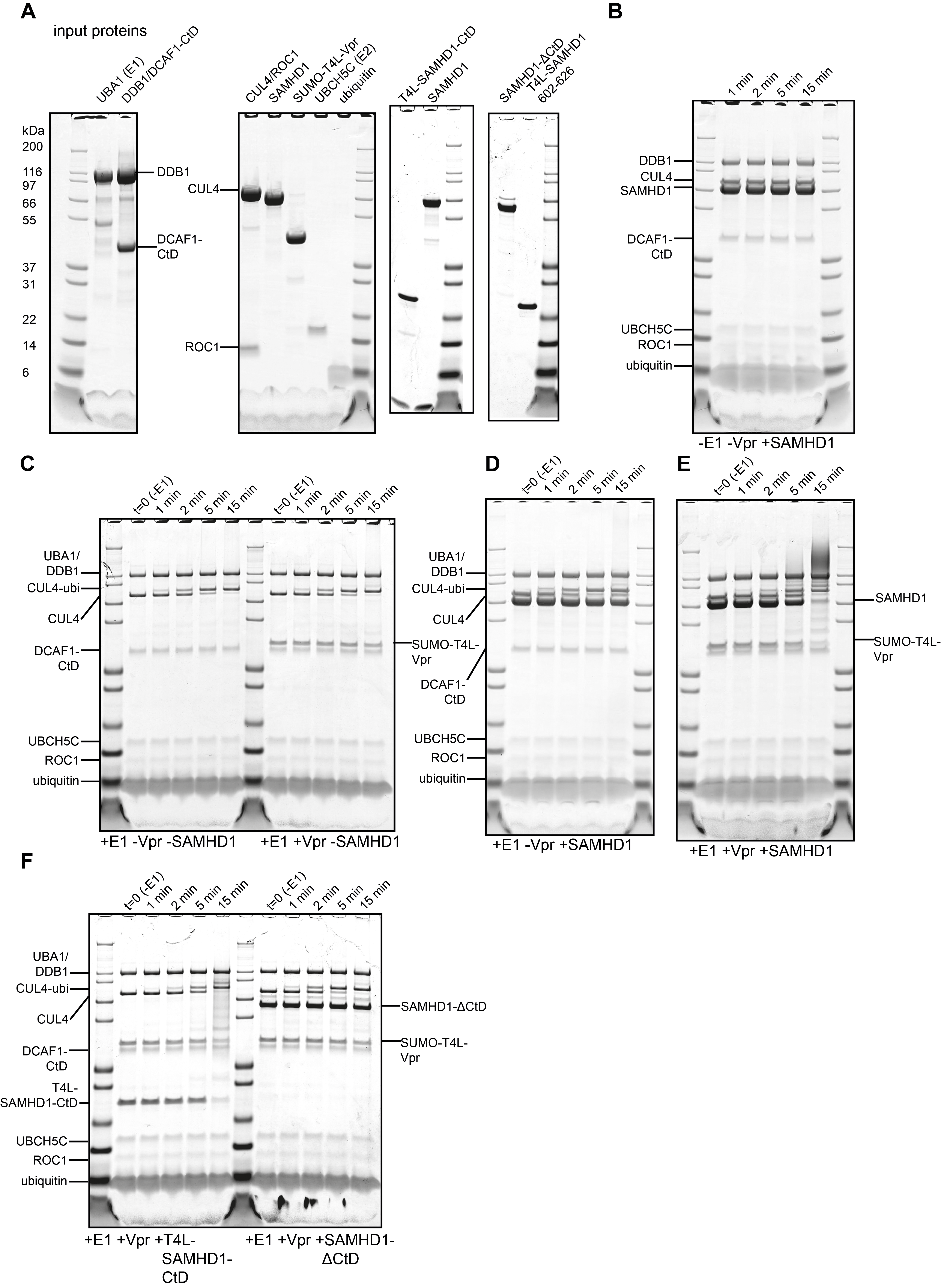

Supplement: S2 Fig — (A) SDS-PAGE of individually purified protein components used in the in vitro ubiquitylation reactions. (B, C) Control reactions in the absence of indicated components. (D-F) Uncropped gels of reactions shown in Fig 1D and 1E and 1F and 1G. All reactions were incubated at 37°C for the indicated times, stopped by addition of SDS sample buffer and separated on SDS-PAGE. (TIF) [file ppat.1009775.s002.tif]

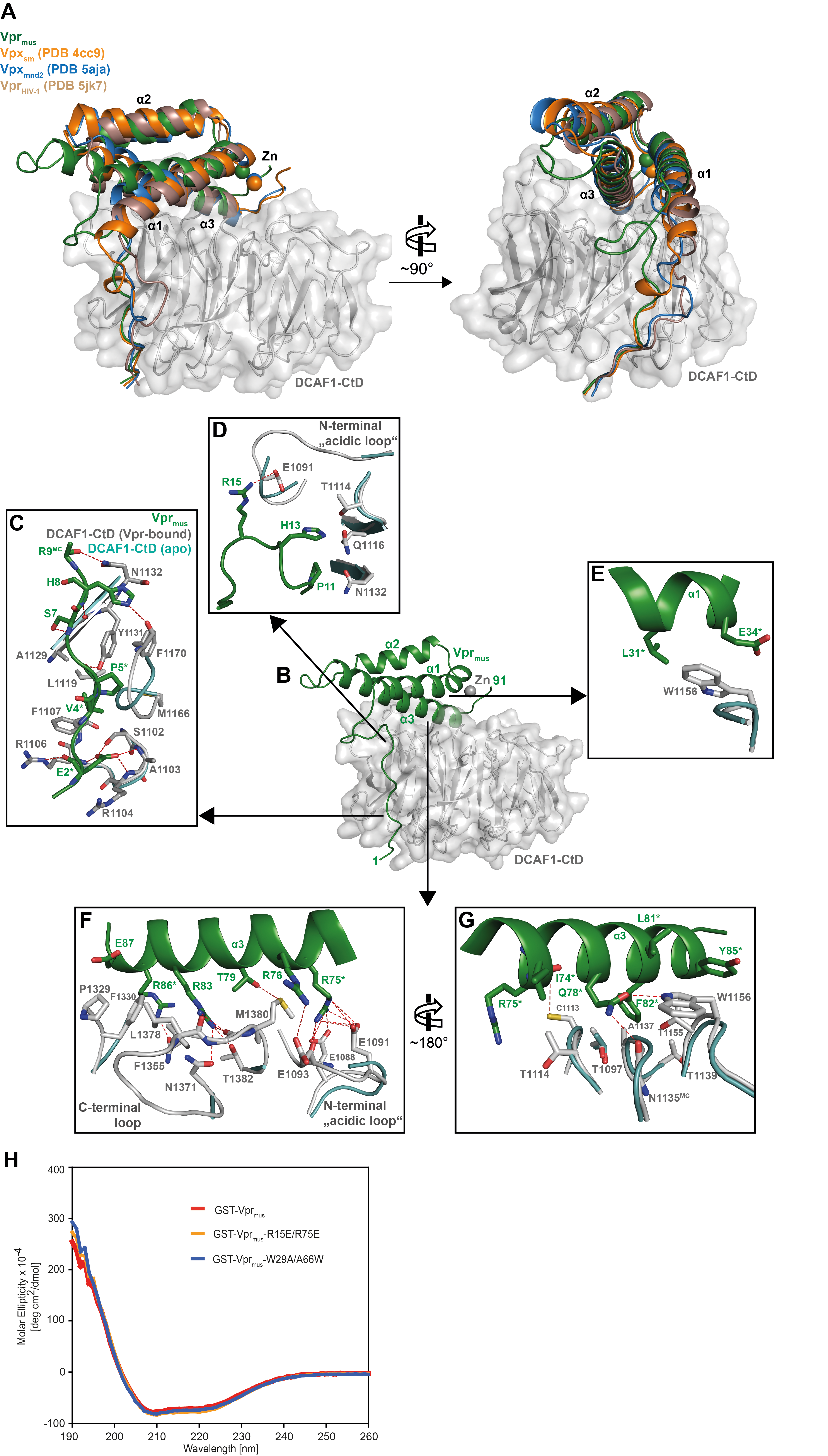

Supplement: S3 Fig — (A) Superposition of the Vprmus (green cartoon)/DCAF1-CtD complex with Vpxsm (orange cartoon, PDB: 4cc9) [50], Vpxmnd (blue cartoon, PDB: 5aja) [51] and VprHIV-1 (light brown cartoon, PDB: 5jk7) [54]. Structures have been aligned with respect to their DCAF1 BP domains but only the DCAF1-CtD from the Vprmus complex is shown for clarity (grey cartoon and semi-transparent surface). (B-G) Details of the DCAF1-CtD/Vprmus interaction. (B) The structure of the complex is shown in the same orientation as Fig 2A, left panel. The insets (C-G) show individual interaction areas in more detail, Vprmus (green), Vprmus-bound DCAF1-CtD (grey) and apo-DCAF1-CtD (light blue). Selected amino acid residues, that make intermolecular interactions, are shown as sticks, and hydrogen bonds/electrostatic interactions as dashed red lines. Vprmus residues with asterisks are type-conserved within all Vpr/Vpx proteins. (H) Circular dichroism (CD) spectra of GST-Vprmus and GST-Vprmus variants R15E/R75E and W29A/A66W. (TIF) [file ppat.1009775.s003.tif]

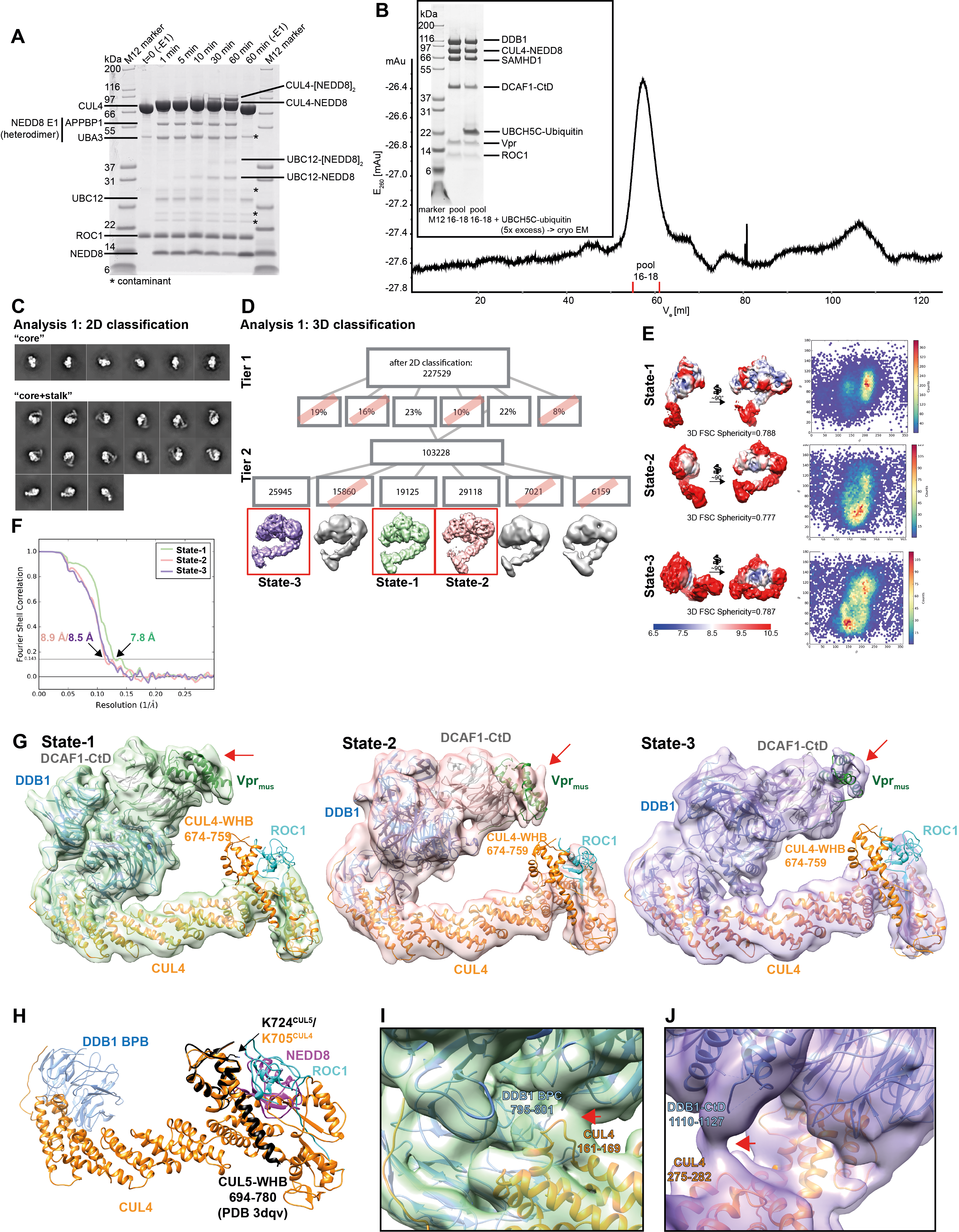

Supplement: S4 Fig — (A) In vitro neddylation of CUL4/ROC1. Protein was mixed with purified neddylation-E1 (APPBP1/UBA3 heterodimer), E2 (UBC12) and NEDD8. The reaction was incubated at 25°C, samples were taken at indicated times, stopped by addition of SDS sample buffer and separated on SDS-PAGE. (B) GF analysis of the CUL4-NEDD8/ROC1/DDB1/DCAF1-CtD/Vprmus/SAMHD1 complex with pooled fractions indicated. A 5x molar excess of UBCH5C-ubiquitin was added before plunge-freezing for cryo-EM experiments, in an attempt to stabilise the assembly. However, no density in any of the reconstructions could be assigned to UBCH5C-ubiquitin, indicating low binding affinity and/or heterogeneity in its mode of binding. (C) 2D class averages depicting either “core” or “core+stalk” classes of analysis 2. (D) 3D sorting tree after 2D classification. Conformational states-1, -2 and -3 are indicated. (E) Local resolution and Euler distribution of states-1, -2 and -3. (F) FSC curves for state-1, -2 and -3 reconstructions. (G) Side-by-side comparison of state-1, -2 and -3 reconstructions, coloured as in Fig 3. Molecular models of the DDB1/DCAF1-CtD/Vprmus crystal structure and CUL4/ROC1 (PDB 2hye) [15] have been fitted as rigid bodies into the volumes and are shown as cartoons. DDB1/DCAF1-CtD/Vprmus is coloured as in Fig 4, CUL4 is coloured yellow and ROC1 cyan. All states show additional density corresponding to SAMHD1-CtD, indicated by the red arrows. (H) Superposition of the neddylated CUL5 C-terminal WHB domain (black cartoon, PDB 3dqv) [56] on the CUL4 WHB (PDB 2hye), coloured as in A. Respective lysine residues, which are covalently modified with NEDD8, are indicated. (I, J) Detailed view of state-1 (I) and state-3 (J) cryo-EM density. Red arrows indicate contacts between CUL4A (orange cartoon) and DDB1 BPA/BPC/CtD (blue cartoon). (TIF) [file ppat.1009775.s004.tif]

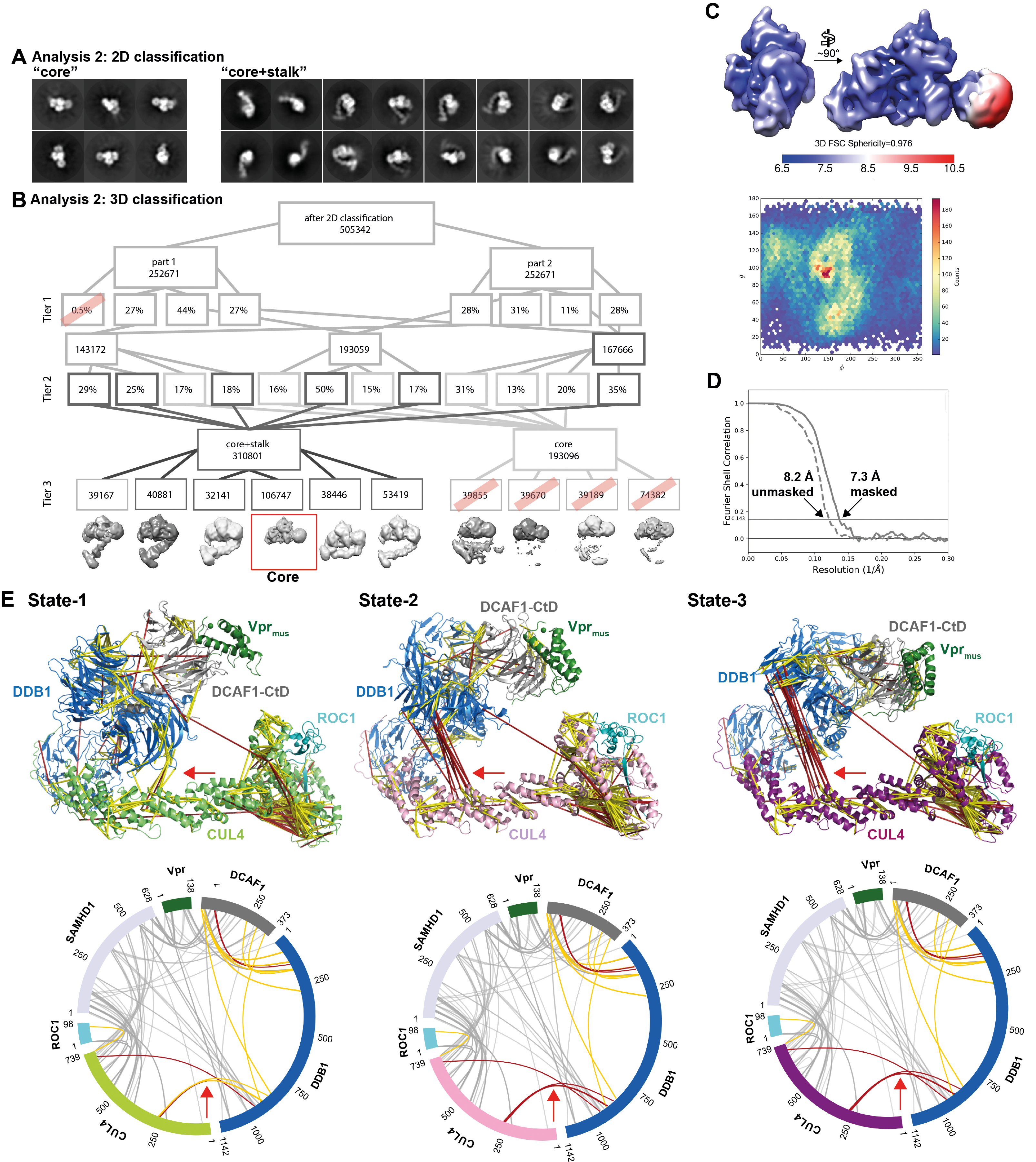

Supplement: S5 Fig — (A) 2D class averages depicting either CRL4-NEDD8DCAF1-CtD/Vprmus/SAMHD1 “core” or “core+stalk” classes of analysis 1. (B) Sorting tree after 2D classification. In Tier 3, the core reconstruction was identified, containing 106,747 particle images (red box). (C) Local resolution of the core reconstruction after refinement, indicating a resolution range from 6.5 Å in the hydrophobic interior of DDB1 to 10.5Å in the DDB1 BPB domain. Below, the Euler distribution is shown. (D) FSC curve of the core reconstruction after refinement. (E) Upper panel: CRL4DCAF1-CtD/Vprmus/SAMHD1 cross-links, identified by CLMS, mapped on molecular models representing state-1, -2 and -3. Satisfied crosslinks (<25 Å) are coloured yellow, violated crosslinks red. Red arrows indicate a subset of cross-links between DDB1 and CUL4, whose distance restraints are satisfied in state-1, and increasingly violated in states-2 and -3. Lower panel: circle plot of CLMS data for states-1, -2 and -3, using the same colour scheme as in the upper panel. Grey lines represent crosslinks between residues that are not present in the molecular models. Only crosslinks between subunits are displayed. (TIF) [file ppat.1009775.s005.tif]

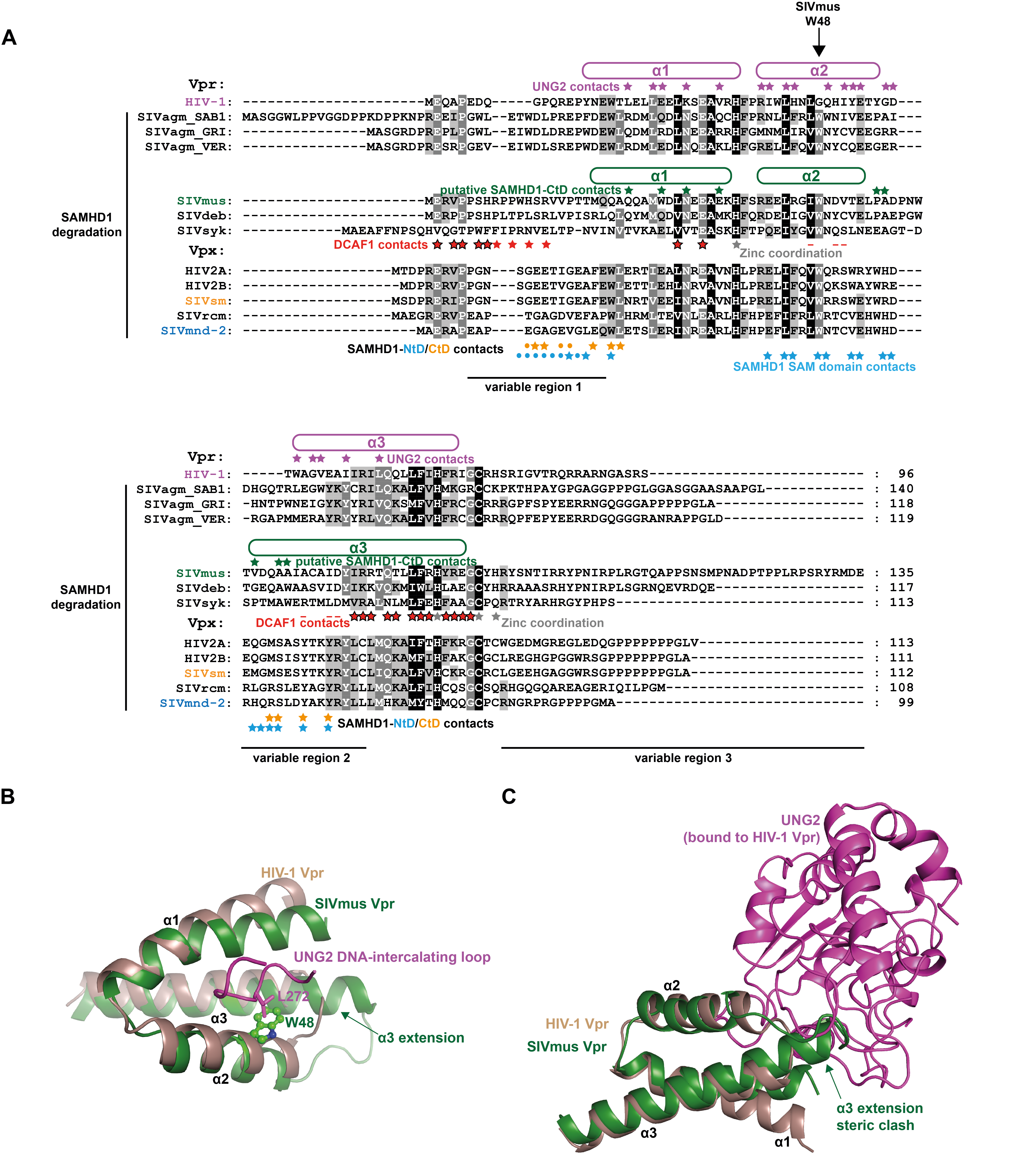

Supplement: S6 Fig — (A) Sequence alignment of indicated Vpr and Vpx proteins. Helices are indicated by the boxes above the amino acid sequences for VprHIV-1 (pink) and Vprmus (green). VprHIV-1 side chains involved in UNG2-binding are indicated with pink asterisks. Vprmus side chains putatively involved in SAMHD1-CtD-binding are indicated by green asterisks. Vpxsm side chains targeting SAMHD1-CtD are indicated with orange asterisks, and Vpxmnd2 side chains contacting N-terminal SAMHD1 domains are highlighted with blue asterisks. Red symbols mark Vprmus side chains involved in DCAF1-CtD-binding. The non-outlined symbols indicate DCAF1-CtD-contacting side chains unique to Vprmus, and dashes show DCAF1-binding side chains, which are in contact with DCAF1-CtD in other Vpr/Vpx structures, but not in Vprmus. Grey asterisks mark Vpr/Vpx side chains involved in zinc coordination. (B) Structural alignment of VprHIV-1 (PDB 5jk7 [54], light brown) in complex with UNG2 and Vprmus (green). Protein backbone is shown in cartoon representation. For clarity, only the DNA-intercalating loop of UNG2 is shown (pink), which inserts into a hydrophobic pocket created by the VprHIV-1 helix bundle. Note the steric clash between UNG2 side chain L272 and Vprmus residue W48 in the structural superposition. (C) Alternative view of the structural alignment of VprHIV-1 (light brown) in complex with UNG2 (pink) and Vprmus (green). Note the steric clash between UNG2 and the extended Helix-3 of Vprmus. (TIF) [file ppat.1009775.s006.tif]
