# Supplementary material for: Structural insights into Cullin4-RING ubiquitin ligase remodelling by Vpr from simian immunodeficiency viruses
Source: PLoS Pathog. 2021 Aug 2;17(8):e1009775. doi: 10.1371/journal.ppat.1009775 (PMC8360603; doi:10.1371/journal.ppat.1009775)
Supplement: S1 Table — *Numbers in parentheses account for the high-resolution shell, **defined in [124]. (PDF) [file ppat.1009775.s007.pdf]

Sample	DDB1/DCAF1-CtD	DDB1/DCAF1-CtD/T4L-Vpr_{mus} 1-92	
PDB code	6zue	-	6zx9
Data collection			
Space group	I222	$P2_12_12$	$P2_12_12$
Cell dimensions			
a, b, c (Å)	117.38, 153.63, 223.16	266.91, 95.94, 99.35	265.90, 95.54, 98.35
α, β, γ (°)	90, 90, 90	90, 90, 90	90, 90, 90
Resolution range (Å)	50.00 (3.28)* – 3.09	50.00 (3.83) – 3.61	79.07 (2.56) – 2.52
R_{merge} (%)	8.8 (120.0)	32.9 (136.1)	9.9 (162.6)
$CC_{1/2}$**	99.9 (85.3)	100.0 (35.4)	99.6 (48.0)
I/σ(I)	13.0 (1.4)	5.9 (1.3)	9.8 (1.1)
Completeness (%)	96.8 (97.1)	99.3 (96.2)	99.9 (99.8)
Redundancy	4.8 (4.9)	6.4 (6.1)	6.6 (6.7)
Refinement			
Resolution range (Å)	48.60 (3.18) – 3.09	-	79.07 (2.55) – 2.52
No. reflections	35922 (2815)	-	84808 (2880)
R_{work}/R_{free} (%)	22.0/27.9 (35.9/43.5)	-	21.6/26.1 (41.1/44.6)
No. atoms			
Protein	11279	-	13556
Ligand/ion	-	-	49
Water	10	-	253
B-factors			
Protein	114.6	-	75.5
Ligand/ion	-	-	77.2
Water	71.7	-	54.9
R.m.s. deviations			
Bond lengths (Å)	0.004	-	0.004
Bond angles (°)	0.759	-	0.794
