# Supplementary material for: Structural insights into Cullin4-RING ubiquitin ligase remodelling by Vpr from simian immunodeficiency viruses
Source: PLoS Pathog. 2021 Aug 2;17(8):e1009775. doi: 10.1371/journal.ppat.1009775 (PMC8360603; doi:10.1371/journal.ppat.1009775)
Supplement: S2 Table — (PDF) [file ppat.1009775.s008.pdf]

Restriction enzyme, insert	Sequence	Destination plasmid (restriction enzymes)
NcoI hsDCAF1 1046 fw.	ggcCCATGGCGccaataaactttacgtcaaggc	pTriEx-6 (NcoI/SacI)
SacI hsDCAF1 1396 rev.	ggcGAGCTCctctgccagacgctgcctgcc	
XmaI T4L (E11H) 2 fw.	ggcCCCGGGaacatttttgaaatgctgcgtattgatg	pHisSUMO (XmaI/NotI)
NotI T4L (E11H) 164 rev.	ggcGCGGCCGCcaggtttttataggcatcccatgtg	
AgeI rhSAMHD1 1 fw.	atattACCGGTatgcagcaagccgactcc	pHisSUMO (XmaI/NotII)
NotI rhSAMHD1 583 rev.	taattGCGGCCGCTTAatcctgaggcttggtgaaatttc	
NotI rhSAMHD1 626 rev.	taattGCGGCCGCTTActttgggtcatctttaaaaagc	
NotI rhSAMHD1 582 fw.	ggcGCGGCCGCAcaggatggtgatgttattgcacc	pHisSUMO-T4L (E11H) (NotI/SacI)
SacI rhSAMHD1 626 rev.	ggcGAGCTCTTAttatttcggatcatctttaaacagctg	
XmaI Vpr_{mus} 1 fw.	ggcCCCGGGatggaacgtgttccgcctagcc	pET49b (XmaI/XhoI)
XhoI Vpr_{mus} 135 rev.	ggcCTCGAGTTAttattcatccatacgataacggctc	
NotI Vpr_{mus} 1 fw.	ggcGCGGCCGCAatggaacgtgttccgcctagcc	pHisSUMO-T4L (E11H) (NotI/SacI)
SacI Vpr_{mus} 92 rev.	ggcGAGCTCTTATTAgcggtgataacaaccttcacgataatg	
Vpr_{mus} R15E fw.	GGCATAGCGAAGTTGTTCCGACCACC	pET49b-Vpr_{mus}
Vpr_{mus} R15E rev.	GGTCGGAACAACTTGGCTATGCCAAGG	
Vpr_{mus} R75E fw.	GATTATATTGAACGTACCCAGACCCTGCTG	
Vpr_{mus} R75E rev.	GTCTGGGTACGTTCAATATAATCAATGGCAC	
Vpr_{mus} W29A fw.	GCACAGCAGGCCATGGCGGATCTGAATGAAGAAGCA	
Vpr_{mus} W29A rev.	TTCTTCATTCAGATCCGCCATGGCCTGCTGTGCCTG	
Vpr_{mus} A66W fw.	GGACCGTTGATCAGGCATGGATTGCATGTGCCATTGATTATATTC	
Vpr_{mus} A66W rev.	CAATGGCACATGCAATCCATGCCTGATCAACGGTCCAATTC	
NdeI ROC1 1 fw.	ggcCATATGgcggcagcgatggatgtgg	pRSF-Duet-1 (NdeI/XhoI)
XhoI ROC1 108 rev.	ggcCTCGAGCTActagtgcccatacttttggaattc	
BamHI hsCUL4A 2 fw.	ggcGGATCCGgcggacgaggccccgcgg	pRSF-Duet-1-ROC1 (12-108) (BamHI/NotI)
NotI hsCUL4A 759 rev.	ggcGCGGCCGCTCAtcaggccacgtagtggtactgattc	
BamHI UBCH5C 1 fw.	ggcGGATCCatggcgctgaaacggattaataag	pGex6P1 (BamHI/NotI)
NotI UBCH5C 147 rev.	ggcGCGGCCGCTCAcatggcatacttctgagtcc	
