# Supplementary material for: Structural insights into Cullin4-RING ubiquitin ligase remodelling by Vpr from simian immunodeficiency viruses
Source: PLoS Pathog. 2021 Aug 2;17(8):e1009775. doi: 10.1371/journal.ppat.1009775 (PMC8360603; doi:10.1371/journal.ppat.1009775)
Supplement: S3 Table — (PDF) [file ppat.1009775.s009.pdf]

**Protein**	**UniProt ID**	**Vector**	**Expression system**
Homo sapiens (hs) DDB1<br>(full length (fl))	Q16531	pAcGHLT-B	Sf9
hsDCAF1-CtD<br>(residues 1046-1396)	Q9Y4B6	pTri-Ex-6	Sf9
Macaca mulatta<br>(Rhesus macaque, rh) SAMHD1<br>(fl)<br>(ΔCtD, residues 1-583)	G7N4W9	pHisSUMO	E. Coli Rosetta 2 (DE3)
T4L(variant E11H)-rhSAMHD1-CtD<br>(residues 582-626)	G7N4W9	pHisSUMO	E. Coli Rosetta 2 (DE3)
SIVmus Vpr (WT and variants R15E/R75E; W29A/A66W)<br>(fl)	A4UDG5	pET49b	E. Coli Rosetta 2 (DE3)
T4L(variant E11H)-SIVmus Vpr (residues 1-92)	A4UDG5	pHisSUMO	E. Coli Rosetta 2 (DE3)
hsCullin4A (CUL4A) (residues 38-759)<br>hsROC1 (residues 12-108)	CUL4A: Q13619<br>ROC1: P62877	pRSF-Duet-1 (Co-expression)	E. Coli Rosetta 2 (DE3)
Mus musculus (mm)<br>mmUBA1<br>(fl)	Q02053	pET28	E. Coli Rosetta 2 (DE3)
hsUBCH5C<br>(fl)	P61077	pGex6P1	E. Coli Rosetta 2 (DE3)
hsUBA3<br>(fl)<br>hsAPPBP1<br>(fl)	UBA3: Q8TBC4-2<br>APPBP1: Q13564	pOPC (Co-expression)	E. Coli Rosetta 2 (DE3)
hsUBC12<br>(fl)	P61081	pGex6P2	E. Coli Rosetta 2 (DE3)
