# Supplementary material for: Structural insights into Cullin4-RING ubiquitin ligase remodelling by Vpr from simian immunodeficiency viruses
Source: PLoS Pathog. 2021 Aug 2;17(8):e1009775. doi: 10.1371/journal.ppat.1009775 (PMC8360603; doi:10.1371/journal.ppat.1009775)
Supplement: Attachment — Submitted filename: 210625_letter_editor.pdf [file ppat.1009775.s010.pdf]

CHARITÉ

ChartitéCentrum für Grundlagenmedizin

Charité | Campus Mitte | 10117 Berlin

To
Professor Michael Malim
Editor-in-Chief
PLOS
Carlyle House
Carlyle Road
Cambridge, CB4 3DN
United Kingdom

Institut für Medizinische Physik und Biophysik

Dr. David Schwefel

Charitéplatz 1 | 10117 Berlin
Interne Geländeadresse: Virchowweg 6 | CCO
Ebene 03 Raum 315

T: +49 30 450 524215
F: +49 30 450 524952
david.schwefel@charite.de
http://biophysik.charite.de

Berlin, June 25, 2021

Revised manuscript PPATHOGENS-D-21-00839

Dear Professor Malim,

I would like to thank the Reviewers of our submission PPATHOGENS-D-21-00839 for their valuable comments, and the editorial board for the opportunity to address the minor points raised by the Reviewers. We have modified the manuscript according to these recommendations. Detailed replies to the Reviewers' comments and the changes made to the manuscript are provided in the following. Altogether, I am positive that the revised version of the manuscript addresses the Reviewers' points appropriately, and that it now fully qualifies for publication in PLOS Pathogens. I am very much looking forward to your reply.

With best wishes,

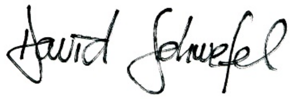

Detailed replies to the Reviewer's comments

Reviewer #1:

Sequence alignment of HIV-1 Vpr, SIVmnd Vpx, SIVsm Vpx, and SIVmus Vpr can be incorporated with Fig. 6, highlighting utilization of different residues at distinct location for specific substrate recruitment, and common or the same residue types for DCAF1 interaction based on the published and current data set. S6 Fig contains such information, however it is too crowded to appreciate the message.

We thank the reviewer for the helpful comment. An appropriate sequence alignment has been included in Fig. 6 and the legend amended accordingly.

Reviewer #2:

1) It is not clear whether dGTP was used during structural analyses. Plus, it is also not clear which form of SAMHD1 is expected in the models presented in Figure 7. If dGTP was not used in any of both biochemical and structural analyses, SAMHD1 dimers could be the form that existed during the analyses. This could be an important issue, particularly because the molecular behaviors of the C-T domain could be significantly different between tetramers (with dGTP) and dimers (without dGTP).

We thank the reviewer for pointing out this omission. We have now expanded the discussion section on Page 18-19 (starting at line 476) around the SAMHD1 oligomerization to state that no exogenous nucleotides were added during purification or any of the follow-up experiments. The text has been amended in the methods section on Page 25 (lines 678-680) and in the Fig 7 legend to also clarify the SAMHD1 oligomeric state we expect under our experimental conditions. Additionally, we now also discuss on Page 18-19 (from line 476) the general implications of SAMHD1 oligomeric state for CRL4-mediated ubiquitylation, and possible ramifications of SAMHD1-CtD sequestration by Vpx/Vpr for SAMHD1 tetramer formation and catalytic activity.

2) It is likely that there is a significant molar ratio difference in infected macrophages between SAMHD1 and Vpr (or Vpx) carried by an infecting virion: SAMHD1 is highly abundant in macrophages whereas Vpr molecule numbers carried by a single virion should be much limited per cell. Therefore, it is not clear whether the model presented in Figure 7 can postulate any scenarios that may explain the degradation dynamics imposed by the vastly different molar ratios between SAMHD1 and Vpr per infected macrophage. Discussion on this issue will be helpful.

Upstream of proteasomal degradation, poly-ubiquitylated substrates are first unfolded by the ATPase p97, and in this way extracted from the substrate receptor. This mechanism thus allows for multiple rounds of SAMHD1 ubiquitylation and degradation by a single Vpr-modified CRL4 assembly. This is indeed an important conceptual point and a paragraph describing this mechanism and how it pertains to the Vpr-SAMHD1 stoichiometric mismatch has now been added to the discussion on Page 19 (starting at line 511).

3) It remains somewhat unclear whether free Vpr captures SAMHD1 first and then binds to DCAF1 in the complex, or Vpr binds to DCAF1 in the complex first and then captures SAMHD1. Possibly the SAMHD1-DCAF1 binding could be more stationary while Vpr binding to SAMHD1 is rather transient. Discussion on this issue will be helpful.

Our *in vitro* reconstitution analyses, though not quantitative, indicate that Vpr_{mus} can engage in equally stable complexes with either SAMHD1 or DCAF1-CtD, at least on the timescale of a gel filtration experiment. A more thorough analysis of binding kinetics to determine on- and off-rates would be necessary to probe this much more quantitatively and we feel that unfortunately this type of study in a complex system is beyond the scope of the present work. However, it is very possible that the order of binding events might also be dictated by subcellular localization. Therefore, we have now added a section into the Discussion on Page 16 (starting at line 397) that describes the scenario of $CRL4^{DCAF1}$ recruitment of Vpr and subsequent capture of SAMHD1.

4) The Vpr proteins that carry anti-SAMHD1 activity has been considered as "ancient" Vpr before the gene duplication to create Vpx, leading to its loss of anti-SAMHD1 function. This point needs to be addressed in Introduction for giving a better definition of "hybrid' Vpr.

On the Reviewer's suggestion, additional sentences have now been inserted on Page 5 and 6 of the Introduction (starting at line 107) to describe the perceived order of events leading to Vpr neo-functionalization and Vpr/Vpx gene duplication.

Summary of changes made to the manuscript

1. Line 5: Middle initial has been inserted
2. Lines 8-9: Affiliation has been corrected
3. Lines 106-117: Modifications addressing point 4) from Reviewer #2
4. Line 371: Fig 6E reference added, addressing the recommendation from Reviewer #1
5. Lines 397-406: Modifications addressing point 3) from Reviewer #2
6. Lines 467-523: Modifications addressing points 1) and 2) from Reviewer #2; additional modifications regarding referencing of Fig 7 panels, which have been re-arranged
7. Lines 678-680: Modifications addressing point 1) from Reviewer #2
8. Line 848: Data availability statement has been removed because it already has been added online in the manuscript tracking system
9. Lines 1288-1293: Modifications addressing the recommendation from Reviewer #1
10. Lines 1298-1305: Modifications addressing point 1) from Reviewer #2
11. Supplemental information captions have been added to the end of the manuscript file
